# A Single Kernel-Based Approach to Extract Drug-Drug Interactions from Biomedical Literature

**DOI:** 10.1371/journal.pone.0048901

**Published:** 2012-11-01

**Authors:** Yijia Zhang, Hongfei Lin, Zhihao Yang, Jian Wang, Yanpeng Li

**Affiliations:** College of Computer Science and Technology, Dalian University of Technology, Dalian, Liaoning, China; Indiana University, United States of America

## Abstract

When one drug influences the level or activity of another drug this is known as a drug-drug interaction (DDI). Knowledge of such interactions is crucial for patient safety. However, the volume and content of published biomedical literature on drug interactions is expanding rapidly, making it increasingly difficult for DDIs database curators to detect and collate DDIs information manually. In this paper, we propose a single kernel-based approach to extract DDIs from biomedical literature. This novel kernel-based approach can effectively make full use of syntactic structural information of the dependency graph. In particular, our approach can efficiently represent both single subgraph topological information and the relation of two subgraphs in the dependency graph. Experimental evaluations showed that our single kernel-based approach can achieve state-of-the-art performance on the publicly available DDI corpus without exploiting multiple kernels or additional domain resources.

## Introduction

In general, a DDI occurs when one drug influences the level or activity of another drug. These DDIs in many ways affect the overall effectiveness of the drug and can sometimes pose a risk of serious side effects to patients [1], [2]. Therefore, the detection of DDIs is crucial for both patient safety and health care cost control. Although health care professionals are supported by different DDI databases, the update periods of these databases are generally three years. Therefore, these databases are rarely complete [3]. Since drug interactions are frequently reported in clinical pharmacology journals and technical reports, a major source of detecting DDIs is the exponential increase in biomedical literature [4]. Thus, the automatic approach of extracting DDIs from biomedical literature can greatly contribute to the management of DDIs and allow scientists and curators early access to new discoveries.

Most biomedical relation extraction corpora have focused on genetic and protein interactions [5], such as BioInfer [6], GENIA [7] and AImed [9], rather than DDIs. Segura-Bedmar et al. created the first annotated DDI corpus [8], which provided an opportunity to use machine learning to automatically extract DDIs. In addition, the DDI Extraction Challenge 2011[8] has attracted more research interests.

One major methodology of relation extraction is pattern engineering, which adopts specific types of patterns or matching rules as the core relation discovery operation [10], [11]. The patterns are mainly represented in the form of sequences of words or syntactic constituents. Blaschke et al. [12] built a set of lexical rules based on clue words. Ono et al. [13], taking into account the surface clues and part-of-speech (POS) rules, defined a group of lexical and syntactic interaction patterns for biomedical relation extraction. Fundel et al. [14] developed a RelEx system for relation extraction, which is based on more syntactic rules. However, the pattern forms are too rigid to capture semantic/syntactic paraphrases or long-range relations. Therefore, these pattern-based methods generally suffer from low recall rates.

Alternatively, with the public availability of large annotated corpora, machine learning methodology has recently become a dominant approach for relation extraction tasks. Although relationships generally involve three or more entities, most of the existing approaches in relation extraction have focused on the extraction of binary relationships, such as DDIs and protein-protein interactions (PPIs). Thus, the methodology of machine learning generally tackles the relation extraction as a classification problem. The major challenge is how to supply the learner with the semantic/syntactic information to distinguish between interactions and non-interactions [15].

**Figure 1:**
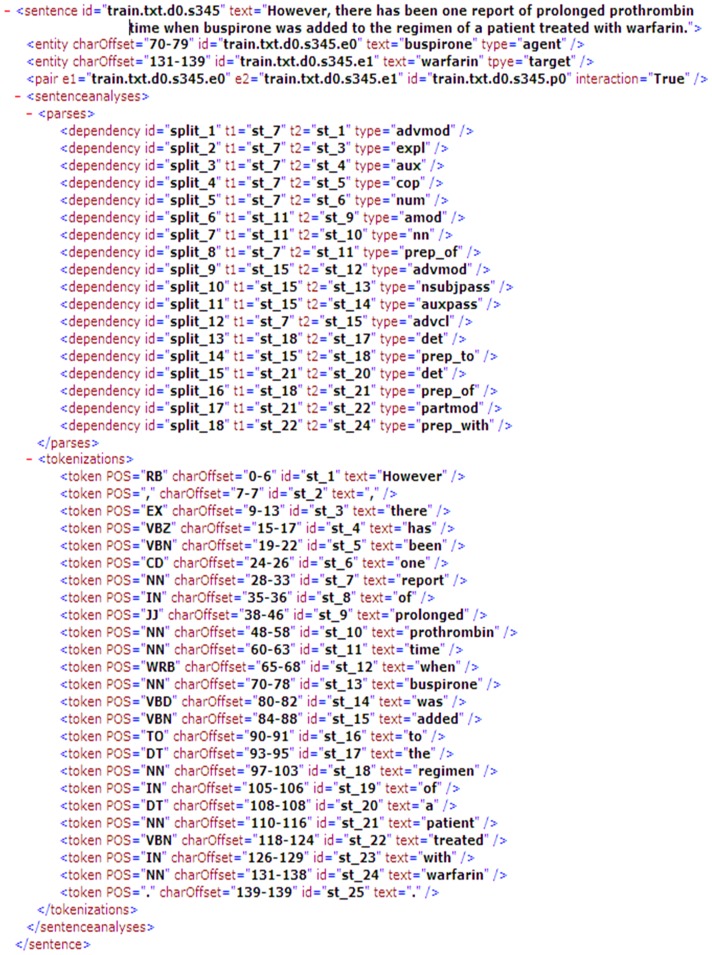
A sentence on the DDI corpus which underwent parsing with the Charniak and Lease parser.

Recent studies [1], [2], [14]–[19] have shown that the dependency graph and syntactic parse tree of a candidate sentence carry vital information for relation extraction tasks if their accuracy is guaranteed. Therefore, approaches such as subsequence kernels [16], [17], tree kernels [18] and shortest path kernels [19] have been proposed and successfully used for relation extraction. The basic idea behind kernel methods is to map the dependency graph or syntactic parse tree into a suitable feature space. Unfortunately, due to the powerful expressiveness of graphs, defining appropriate graph kernel functions has proved difficult [20]. In order to control the complexity of kernel methods, existing kernel methods generally exploit limited information of the dependency graph representing the sentence structure. For instance, the walk-weighted subsequence kernel [17] matches the e-walk and v-walk on the shortest path of the dependency graph, which can only represent the semantic/syntactic information of the shortest path. The tree kernel [18] can represent tree structure, but it is still not enough to completely represent all the semantic/syntactic information of the dependency graph. The all-path graph kernel [15] only computes the basic label of each node and neglects the contiguous structure of the node. The NH kernel [21] can represent the single subgraph topological information, but cannot represent the relation information of different subgraphs in the whole dependency graph. In an effort to improve the performance of these kernel methods, researchers have concentrated on combining them. Miwa et al. [22] proposed the composite kernel, which combines multiple kernels: the all-path kernel, the bag-of-words kernel and the subset tree kernel. A similar approach was used by Yang et al. [23] who proposed a weighted multiple kernel learning-based approach including the feature-based kernel, tree kernel, all-path kernel and POS path kernel. In particular, the DDI Extraction Challenge 2011 [8] showed that approaches based on multiple kernels achieved better results than other approaches.

**Figure 2:**
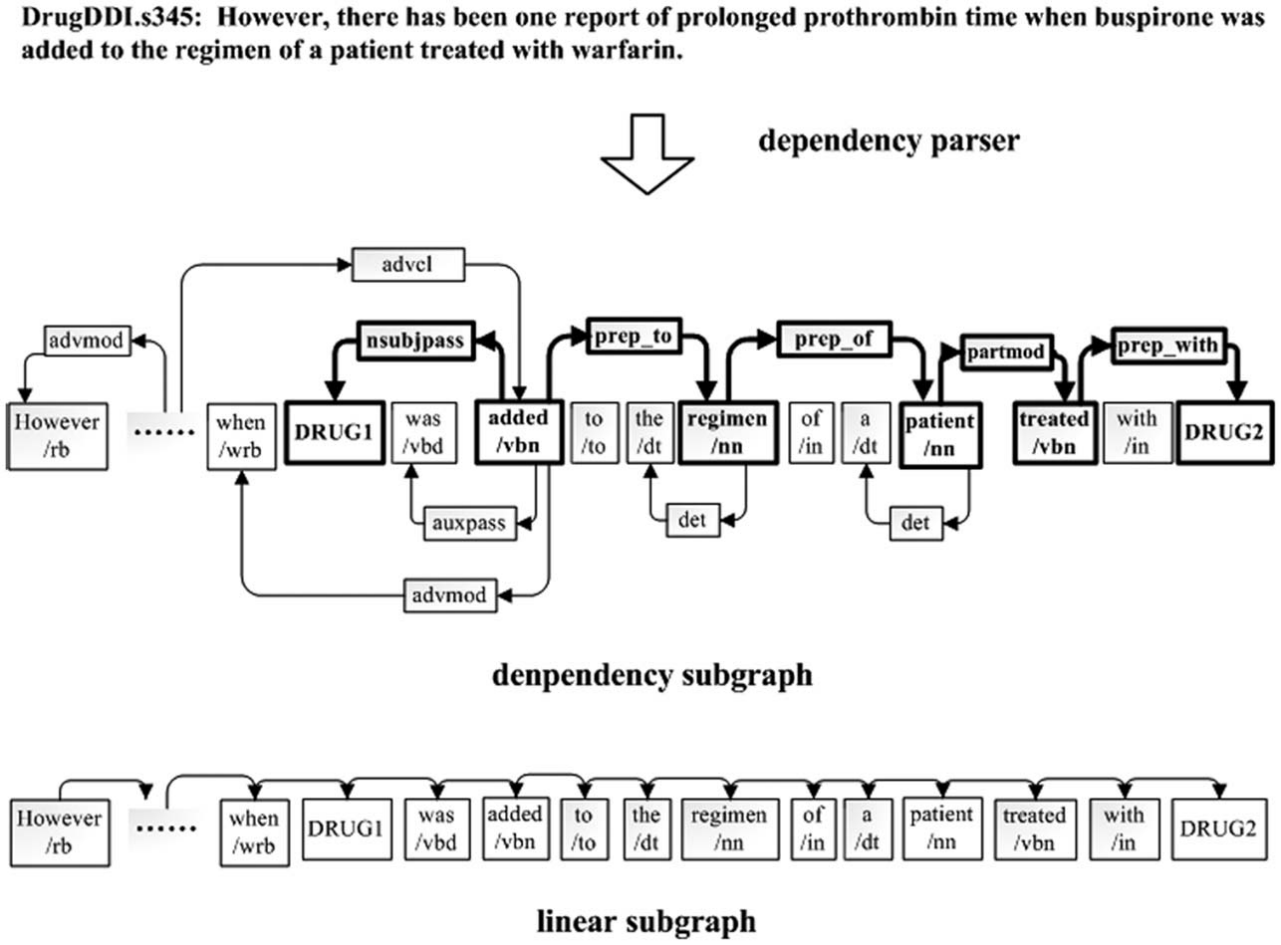
Graph representation of a sentence.

In this paper, we propose the hash subgraph pairwise (HSP) kernel-based approach for DDIs extraction tasks. We show that a single kernel-based approach can achieve state-of-the-art performance without exploiting multiple kernels. Compared to the existing kernel approaches, the HSP kernel can efficiently represent more structural information of the dependency graph. Firstly, we represent the dependency structure and linear order of candidate sentence by a graph representation including the dependency subgraph and linear subgraph. Secondly, we construct hierarchical labels for each node of graph and use the hash operation to compute the value of the labels, which can effectively represent the contiguous structure of the subgraph. Generally, the relation between nodes or subgraphs has an impact on the classification of graphs. Thirdly, based on this hypothesis, the HSP kernel maps the graph into the subgraph pairs feature space. In particular, the HSP kernel can set each subgraph pair feature by different weights according to the distance between the subgraph pair. Therefore, the HSP kernel can represent the single subgraph topological information as well as the relation of two subgraphs. Since the whole original sentential structure contains noise for DDIs extraction tasks, we propose a graph pruning method to prune apparently noisy information from the original sentential structure and emphasize the relevant syntactic information. We evaluate the HSP kernel approach on the DDIs Extraction Challenge 2011 task corpus and compare our approach with state-of-the-art approaches.

**Figure 3:**
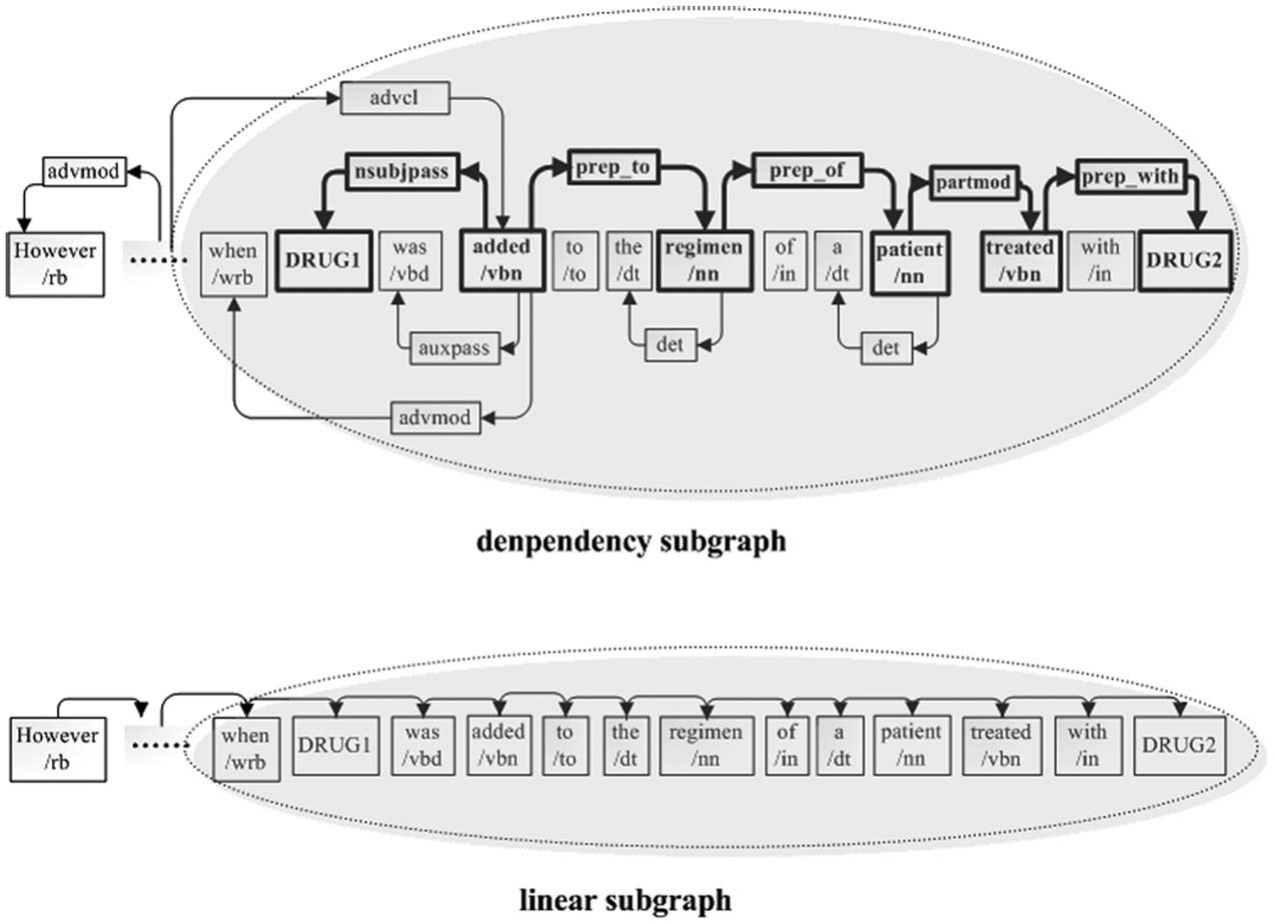
Illustration of pruning the dependency subgraph and linear subgraph.

## Methods

In this section, we first present the graph representation of sentence structure. Then we introduce how to prune the graph representation to remove the noisy information from the original sentential structure. Finally, we describe in details how to use the HSP graph kernel to extract DDIs.

**Figure 4:**
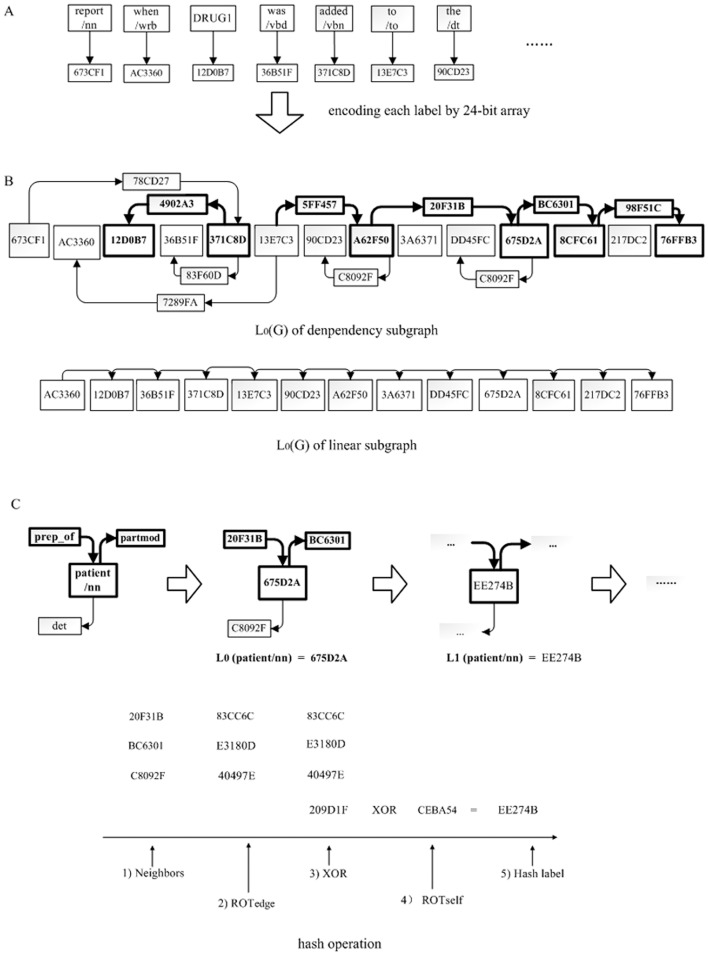
An example of how to compute hierarchical hash labels.

### Graph Representation of Sentence Structure

As in recent studies of DDIs extraction [1], [2], [8], we tackled the task by learning a decision function that determines whether an unordered candidate drug pair has a relevant relationship in a sentence. Recent studies [1], [2], [14]–[19] have shown that biomedical relation extraction can benefit from the dependency graph or syntactic parse tree of candidate sentences. Therefore, the candidate sentence underwent parsing with the Charniak and Lease parser [24] and we then supplemented the syntactic information as the unified format proposed by Pyysalo et al. [25]. Figure 1 shows an example of a candidate sentence (DrugDDI.s345) that contains syntactic information including the dependency relation and POS of each token. Secondly, we represented each sentence as a directed vertex-labeled graph that consisted of a dependency subgraph and linear subgraph, which was similar to previous studies [15], [21]. The dependency subgraph represented the dependency structure of the sentence and the linear subgraph represented the linear order of the sentence. Figure 2 is the graph representation generated from the sentence in Figure 1. In Figure 2, every node has a label for the token or dependency relation. For instance, “treated/vbn” denotes that the text of the token node is “treated” and the POS is “vbn”, whereas “nsubjpass” denotes that the dependency relation of token nodes “DRUG1” and “added/vbn” is “nsubjpass” type. DRUG1 and DRUG2 denote candidate drug names, respectively, and the shortest path between them is shown in bold in the dependency subgraph.

**Figure 5:**
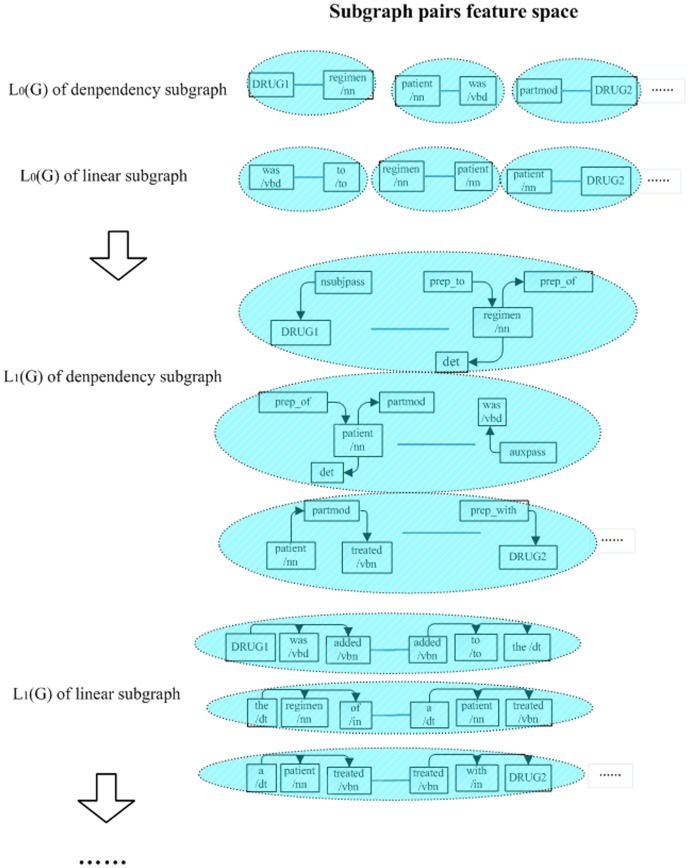
Illustration of subgraph pairs features mapped from 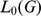, 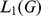,… 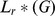
.

### HSP Graph Kernel

In recent years, various kernel methods have been employed for this task. In general, the syntactic structures around the candidate drug pairs contain more valuable information for DDIs tasks in the dependency graph. Therefore, we proposed the HSP kernel for DDIs task based on the all-path graph kernel [15], which can represent the single subgraph topological information as well as the relation of two subgraphs. We first briefly introduced the following related notion:

Let 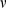 be a set of vertices (or nodes) and 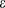 be a set of edges (or links). Then, a graph 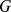  =  (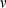, 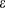) is called a directed graph if 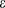 is a set of directed links 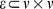.

Definition 1 (Vertex-Labeled Graph) Let 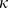 be a set of labels (or attributes) and 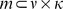 be label allocations. Then, 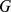  =  (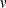, 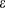, 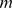) is called a vertex-labeled graph.

Definition 2 (Inner Product) For two 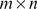 matrices A and B, the inner product is defined as (1).
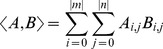
(1)


Firstly, we used a unique binary array consisting of 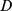-bits (0 or 1) to denote each label of the graph 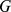, such as 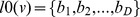, where the constant 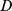 satisfies 
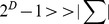
. Therefore, it can represent an unsigned integer value up to 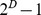, and the node label set 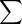 is a finite set of discrete values. Thus, we can obtain a basic bit label 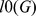 of 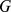. Secondly, we followed previous studies [21], [26] to define the hash operation. Let 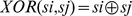 denote the 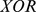 operation between two bit labels 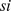 and 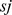, which produces another binary array with each bit representing the 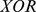 value for each digit. Let 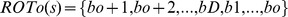 denote the 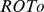 operation for 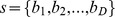 shifts the last 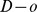 bits to the left by 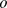 bits, and moves the first 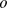 bits to the right end. We can iteratively calculate the hash label for each node using (2), where 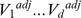 denote the adjacent nodes of 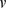. Moreover, we can distinguish between in-coming edge and out-going edge by setting different 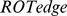 operations. For instance, if the edge 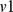 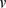 is an in-coming edge of node 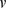, let 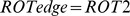, and if the edge 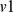 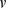 is an out-going edge of node 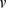, let 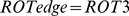.
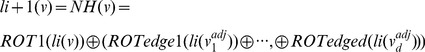
(2)


Let 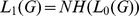 denote the neighborhood hash function to a graph 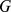. Furthermore, the neighborhood hash function can be applied iteratively as 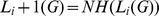, and 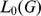, 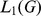, 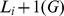, is the hierarchical hash labels of 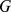. Since the hash operation can aggregate the neighborhood nodes, 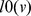 only represents the basic information of node 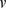, whereas 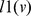 can represent the structural information of the subgraph of radius 1. Similarly, 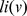 has the capability to represent the structural information of the subgraph of radius 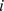. All bit operations such as 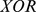 and 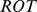 can be done in one clock, if the fixed length 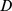 is no more than the bit size of the processor architecture (32 or 64). Therefore, we can efficiently compute the hierarchical hash labels of the whole graph. Finally, based on hierarchical hash labels, the HSP graph kernel is defined as (3), where 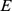 is the adjacency matrix of 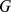 and 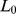, 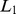
… ,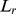 are the hierarchical hash labels of 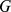.
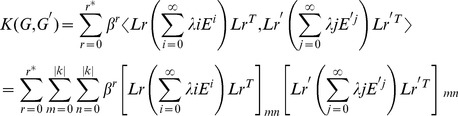
(3)It is well known that each element 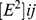 gives the number of walks of length 2 from 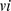 to 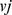. Similarly, each component 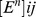 gives the number of walks of length 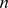 from 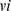 to 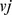. Due to the hierarchical hash labels, 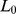, 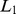
… ,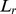 can express the distribution of the contiguous neighbors of each node. The matrix power series 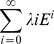 combines the effect of subgraph pairs with different distances. 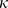 is the set of possible hash labels and 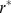 is the upper bound for the number of hierarchy. Hence, the HSP kernel can represent the full graph by mapping the graph into high dimensional subgraph pairs feature space, rather than concentrate only on special types of graphs, such as the tree kernel and string kernel. In particular, the HSP kernel can more effectively represent the relation of subgraphs. 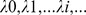 are the weights sequence for subgraph pairs with different distances (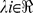; 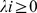). To control the complexity of the HSP kernel, we let 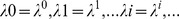 and 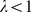, and efficiently calculated the matrix power series using (4). In equation (4), matrix inversion is only the cubic time complexity.



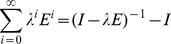
(4)In addition, we set the decay factor sequence 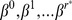 for hierarchical hash labels in (3) to scale the impact of subgraph pairs with different sizes, where 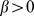.

### Graph Pruning Method

The HSP kernel can accurately compute the contiguous topological information and the relative information of subgraph pairs in a graph. Therefore, the noisy information of graph representation can obviously reduce the performance of the HSP kernel. Unfortunately, previous studies [15], [17], [21] have shown that the whole original sentence structure contains too much noise for biomedical relation extraction tasks. To address this problem, we proposed a pruning method to prune apparently noisy information from the sentence representation and emphasize the relevant syntactic information.

**Table 1:** Statistics of the DDI corpora.

	Training sets	Test sets	Total
Documents	435	144	579
Candidate DDI pairs	23827	7026	30853
Positive DDI pairs	2402	756	3158
Negative DDI pairs	21425	6270	27695

Pruning methods for relation extraction were first introduced by Zhang et al. [27] who suggested seven types of pruning methods [28]. For biomedical relation extraction, the study [15] showed that the shortest path between candidate proteins in the dependency subgraph contained more vital distinguishing information. Bunescu et al. [19] followed this hypothesis and only exploited the shortest path information of the dependency graph. Furthermore, Zhou et al. [29] reported that subtrees enclosed by the shortest path between two entities describe their relation better than other subtrees, even though, in some cases, these subtrees can miss important syntactic structures. However, few approaches have been used to prune the dependency graph for biomedical relation extraction tasks. Next, we introduced the method to prune the graph representation in Figure 2. We divided the graph representation into the dependency subgraph and linear subgraph. For the dependency subgraph, we preserved only the tokens on the shortest path between candidate drug pairs, their direct neighbor tokens and dependency relations between these tokens. For the linear subgraph, we preserved the tokens between candidate drug pairs and the direct neighbor tokens of candidate drugs. Figure 3 shows how to prune the dependency subgraph and linear subgraph. To preserve the vital context, we pruned the syntactic structure out of the shadow region and preserved only the syntactic structure encompassing the candidate drug entities and between them. From Figure 3, it can be seen that we mainly preserved the syntactic structures between “when/wrb” and “DRUG2”, and pruned the syntactic structure of “However, there has been one report of prolonged prothrombin time” in both the dependency subgraph and linear subgraph. Without pruning, all the syntactic structures of the sentence will intricately participate in deciding the candidate DDI. Therefore, the pruning method prunes apparently noisy information as well as emphasizes the relevant syntactic information. For example, without pruning, although the syntactic structure of “However, there…prothrombin time” contains little valuable information, it still participates in predicting the candidated DDI. Instead, after pruning, the classifier can concentrate on the syntactic structure of “when …DRUG2”. In addition, the pruning method can effectively separate features when two or more interactions exist in a sentence.

**Table 2:** Effectiveness of parameterβ.

β	*P*	*R*	*F*	σF	AUC	σAUC
β = 0.2	55.5	64.6	59.7	2.1	88.3	1.8
β = 0.4	56.4	66.5	61.0	2.8	89.5	1.7
β = 0.6	56.8	**68.0**	**61.9**	2.4	90.1	2.2
β = 0.8	57.1	67.4	61.7	2.7	90.6	1.5
β = 1.0	56.7	65.6	60.8	1.8	**91.0**	1.4
β = 1.5	57.3	63.3	60.2	2.0	90.7	1.9
β = 2.0	**57.6**	62.5	59.9	1.7	90.2	1.2

*F*: *F*-score; *P:* precision; *R:* recall. σF andσAUC are the standard deviation of the *F*-score and AUC in cross validation, respectively.

### DDIs Extraction

We next present how to use the HSP graph kernel to extract DDIs from biomedical literature. Firstly, we used a 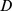-bits binary array to encode each node of the graph representation. To reduce the problematic hash collisions, we chose D = 24 in our experiments. Figure 4A shows the encoding process of the graph representation in Figure 3 and the bit labels are represented by hex forms. Thus, we can obtain the 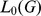 of the dependency subgraph and linear subgraph, as shown in Figure 4B. Secondly, we computed the hierarchical hash labels for each node in the dependency subgraph and linear subgraph. Figure 4C illustrates how to calculate the hash label of the node “patient/nn”. The value of 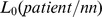 was “675D2A” which only represented the basic token information of the node. However, the value of 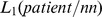 was “EE274B” which represented the contiguous structure information of 1-neighbors. Furthermore, we can obtain the 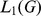 by calculating the whole graph representations from 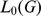. Similarly, we iteratively computed the hierarchical hash labels of 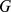, that is 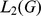, …, 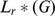. Thirdly, we assigned the same weight 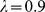 to all edges of the graph representation and computed the similarity of two graph representations using (3).

**Table 3:** Performance of our approach in comparison with other approaches.

Approach	*TP*	*FP*	*FN*	*TN*	*P*	*R*	*F*	*Acc*	*MCC*	*AUC*
WBI [31]	543	354	212	5917	60.5	71.9	**65.7**	91.9	**61.5**	-
Our approach	508	297	248	5973	**63.1**	67.2	65.1	**92.2**	60.8	**92.4**
LIMSI-FBK [32]	532	376	223	5895	58.6	70.5	64.0	91.5	59.5	-
FBK-HLT [33]	529	377	226	5894	58.4	70.1	63.7	91.4	59.2	-
UTurku [34]	520	376	235	5895	58.0	68.9	63.0	91.3	58.4	-
BNBNLEL [36]	420	266	335	6005	61.2	55.6	58.3	91.5	53.6	-
Naive Bayes approach	603	786	153	5484	43.4	**79.8**	56.2	86.6	52.3	84.1
SVM approach	382	346	374	5924	52.5	50.6	51.5	89.8	45.8	87.2

*F: F*-score; *P:* precision; *R:* recall; *Acc:* accuracy; *MCC:* Matthews correlation coefficient.

**Figure 6:**
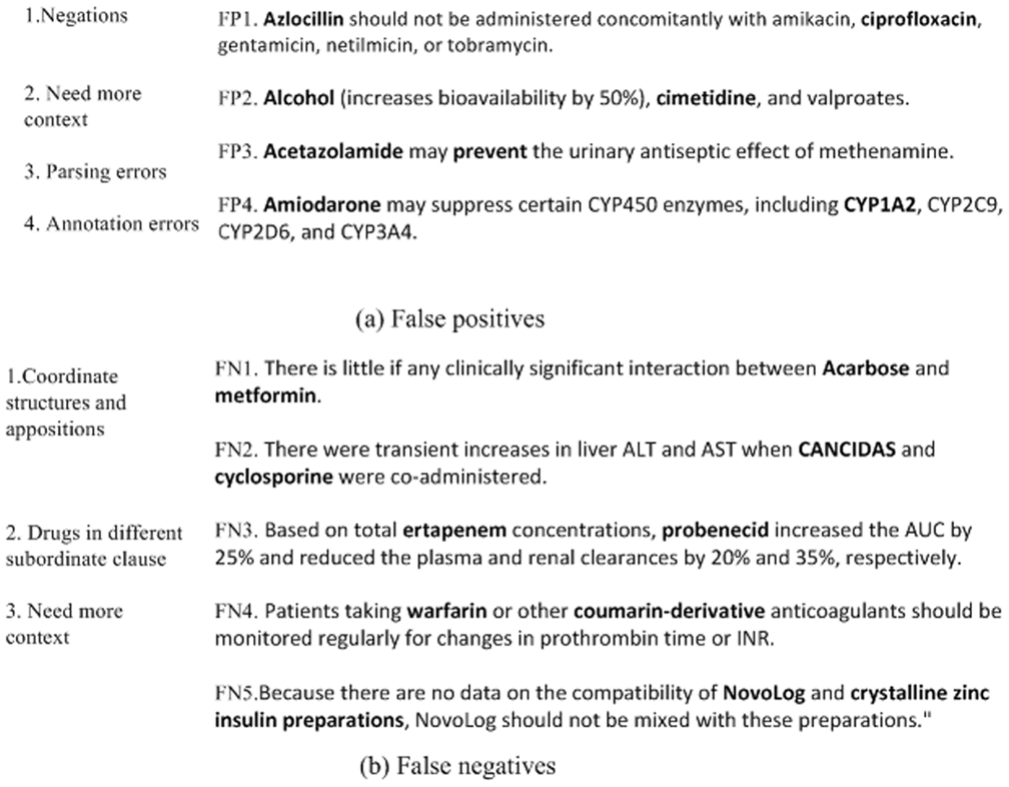
Examples of false negatives and false positives generated by our approach.

In essence, the HSP graph kernel can map the dependency subgraph and linear subgraph into subgraph pairs feature space. In Figure 5, the shadow regions denote the subgraph pairs features which were extracted from the 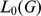, 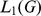,…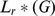. For 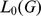, each subgraph only contained one node, and any two subgraphs in the dependency subgraph or in the linear subgraph formed a subgraph pair feature. For instance, the nodes “DRUG1” and “regimen/nn” in 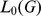 of the dependency subgraph formed a feature “DRUG1”-“regimen/nn”. For 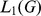, each subgraph contained one central node and its direct neighbor nodes (1-neighbors). In other words, each subgraph can represent the topology information and syntactic information of the region with a radius of 1. We compared the feature space of the HSP kernel with the tree kernel and string kernel. On the one hand, the features extracted from the linear subgraph were similar to the features extracted by the string kernel. On the other hand, the features extracted from the dependency subgraph were more complex than the features extracted by the tree kernel. Therefore, compared with the tree kernel and string kernel, the HSP kernel can map the graph representation into both simple features and complex syntactic features. Moreover, the subgraph pair feature can represent the relation between any two complex syntactic structures in the dependency subgraph or linear subgraph. Obviously, the subgraph pair feature of 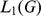 contained much more valuable information than the feature of 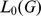. However, with the enlargement of the subgraph, the large-scale subgraph pair feature will cause the system to classify instance in a strict manner, which will generally lead to over-fitting problems. Thus, we should control the upper bound parameter 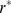 to balance the performance of the HSP kernel for different tasks.

### Experimental Setting

We evaluated our method using DDI Extraction Challenge 2011 corpora [8] which is the first publicly available corpora for DDI extraction tasks. The statistics of the DDI corpora are listed in Table 1, which contains 579 documents and 30853 candidate DDIs pairs. These documents were randomly selected from the DrugBank database, which were split into training sets and test sets. In addition, all sentences in the documents underwent parsing with the Charniak and Lease parser [24], and the syntactic information was added similar to the example shown in Figure 1. The implement of our method with the name DDI_Extraction_Tool.zip is available in Supplementary Information.

To keep our evaluation metrics the same as the DDI Extraction Challenge 2011 task [8], we optimized the parameters of our approach for DDIs extraction tasks by conducting 10-fold cross validation on the training datasets, and then tested the test datasets. This guaranteed the maximal use of the available data and allowed a comparison with the other approaches. We implemented the HSP kernel with the user defined kernel interface of SVM-light (http://svmlight.joachims.org/). Similar to previous studies [15], [21], we empirically estimated the regularization parameters of SVM (C-values) on training datasets.

The majority of DDI extraction system evaluations use the balanced *F*-score measure for quantifying the performance of the systems, which is defined as *F*-score  =  (2*PR*)/(*P+R*), where *P* denotes precision and *R* denotes recall. In addition, we reported the AUC measure [30] and MCC measure [37], which have been recommended for performance evaluation [15], [21], [23], [38].

## Results and Discussion

### Performance on Training Datasets

Firstly, we set the parameter 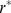 in (3), which is the upper bound for the number of hierarchy. In general, an increase in the hierarchy of hash labels will cause the HSP kernel to compute more large-scale subgraph pairs and the system classifies each instance in a strict and detailed manner. Therefore, when the value of 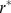 is too large, over-fitting problems will generally occur particularly for biomedical relation extraction tasks. After preliminary experiments, we set 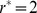 in our experiment.

Secondly, we investigated the effect of decay factor 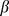 in (3) for DDIs extraction tasks, which can balance the degree of contribution of subgraph pairs with different sizes in the HSP kernel computation. Table 2 shows the evaluation results on the training datasets and the value in bold is the highest value of each column. It is obvious that parameter 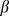 influenced the overall performance of DDIs extraction. The gap between the best and worst *F*-score was 2.2%. In particular, our approach achieved the best performance (an *F*-score of 61.9%), when 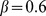. From Table 2, it can be seen that the precision can be improved when 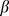 is increased (from 55.5% to 57.6%). Similarly, the recall can be improved significantly when 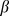 ranged from 0.2 to 0.6. However, when 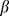 ranged from 0.6 to 2.0, the recall dropped sharply. The main reason for this is that the increase in 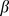 promoted the weights of larger subgraph pairs for the HSP kernel. Since large subgraph pairs contain more syntactic/semantic structural information than small subgraph pairs, this caused our approach to classify each instance in a strict and detailed manner. Therefore, the increase in 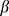 generally contributes to the precision. However, if 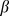 is too large, it will reduce the recall drastically. In addition, our approach achieved best AUC of 91.0% at 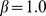, which was similar to the change in *F*-score as a whole. Consequently, we choose 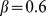 as the optimal parameter for DDIs extraction tasks.

### Performance of our Approach Compared to Other Approaches

We tested our approach on test datasets using optimal parameters. In recent years, some kernel methods have been proposed and successfully applied to biomedical relation extraction. However, most of these studies focused on PPI extraction, and not DDI extraction. Although PPI extraction is similar to DDI extraction, there are some differences between them. For instance, the terminological specificity and the way researchers report their findings in different biomedical domains vary considerably. Therefore, the results from PPI extraction do not necessarily extrapolate to DDI extraction. To evaluate the performance of our approach for DDI extraction, we compared our approach with other approaches (Table 3) which included the top rank approaches reported in the DDI Extraction-2011 Challenge and other simple machine learning approaches, such as SVM and naive Bayes (NB). We built rich features for the SVM and NB approaches and empirically estimated the regularization parameters of SVM (C-values). The rich features consisted of bag-of-words (BOW) features, bigrams features and trigrams features. The NB approach achieved a high recall of 79.8%, but overall both the NB approach and SVM approach were inferior to our approach. For instance, our approach achieved an F-score of 65.1% which was an 8.9% point margin compared to the NB approach and a 13.6% point margin compared to the SVM approach. These results indicated that the common features such as BOW features and word-gram features are not enough for DDI extraction tasks, because the vital syntactic information of the dependency graph cannot be directly mapped into the feature space.

It is known that the combination of multiple kernels is the best option for improving the effectiveness of kernel-based approaches [22], [23]. From Table 3, it can be seen that the WBI approach [31] achieved best performance (an *F*-score of 65.7%, a recall of 71.9% and a MCC of 61.5%), this approach exploits the combination of several kernels and a case-based reasoning system using a voting approach. In addition, the WBI approach also achieved best performance in the DDI Extraction 2011 Challenge. Similarly, the LIMSI-FBK approach [32] and the FBK-HLT approach [33] achieved competitive performances (*F*-score of 64.0% and 63.7%, respectively), and both approaches benefited from compositing several kernels including the MEDT kernel, PST kernel, and SL kernel. In addition, the UTurku approach [34] exploits the domain knowledge such as DrugBank [35], and achieved an *F*-score of 63.0% and a MCC of 58.4%. The BNBNLEL approach [36] constructs rich features and uses random forests to extract DDIs. However, due to a low recall of 55.6%, the BNBNLEL approach only achieved an *F*-score of 58.3% and a MCC of 53.6%. Compared with the above approaches, our single kernel-based approach achieved the best precision (63.1%) and the best accuracy (92.2%). Moreover, our approach achieved an *F*-score of 65.1% and a MCC of 60.8%, which was only slightly inferior to the WBI approach. In particular, our approach was obviously superior to the LIMSI-FBK approach [32] and FBK-HLT approach [33], which are composed of the tree kernel, context kernel and other kernels. This indicated that our approach can more accurately compute and represent the syntactic structural information than other such kernels for DDIs extraction tasks. However, we also note that our approach only achieved a recall of 67.2%, which was far below the multiple kernel-based approaches. This was mainly because the multiple kernel approaches can take into account richer features than the HSP kernel. For instance, as the WBI [31] approach consists of three kernels, it can classify each candidate DDI based on all-paths graph features, shortest path features and shallow linguistic features. Overall, we can only use the HSP kernel to achieve state-of-the-art performance rather than combine several kernels or exploit additional domain resources.

### Error Analysis

Finally, we manually analyzed drug mention pairs which were not correctly classified by our approach. Evaluated using the final test set, our approach made a total of 545 errors, 297 of which were false positives and 248 of which were false negatives. Figure 6 shows the principal causes for the false positives and false negatives generated. The two drugs of each candidate pair are in bold.

The most frequent cause of false positives is our approach was the failure to identify negation expressions. In Figure 6a, FP1 is an example of these false positives. Therefore, a possible approach to improve performance is to introduce a pre-processing step for negation expressions. Another frequent cause of false positives is the DDI cannot be verified without the context. For instance, in Figure 6a FP2, there is very little information in the sentence and we could not verify the interaction between “Alcohol” and “cimetidine” without context. Furthermore, some false positives are caused by corpus errors. In FP3, “prevent” is a verb, but the corpus treats “prevent” as a drug due to parsing error. Moreover, according to the sentence FP4, the DDI between “Amiodarone” and “CYP1A2” should be annotated by “True”, however, the corpus annotates the DDI with “False”.

With regard to false negatives, most errors are caused by coordinate structures and appositions. In Figure 6b, FN1 and FN2 are two examples of these false negatives. Further studies should be performed on coordinate structures and appositions. In addition, it is difficult for our approach to deal with complex sentences in which two drugs are in different subordinate clauses. For example, our approach failed to verify the DDI in FN3. As in false positives, some false negatives are due to the need for more context information. In FN4 and FN5, we could not verify the DDIs without more context information. Therefore, such candidate DDIs should be taken out of the DDI corpus.

## Conclusion

In this paper, we propose a single kernel-based approach to automatically extract DDIs from biomedical literature. To preprocess the dependency graph, we applied a novel pruning method to prune apparently noisy information and emphasize the relevant syntactic information. The experimental results demonstrated that our approach can effectively represent the syntactic structural information of the dependency graph. Furthermore, it is encouraging to see that our single kernel-based approach was comparable to the top rank multiple kernel-based approaches, and achieved state-of-the-art performance. Our major contributions to this research field are:

1. We proposed the HSP kernel approach for DDIs extraction tasks and evaluated our approach on a publicly available DDI corpus.
2. We proposed a novel pruning method to prune apparently noisy information of the dependency graph and emphasize the relevant syntactic information.
3. We evaluated our approach on a publicly available DDI corpus and compared the performance of our approach with state-of-the-art approaches.
4. The experimental results indicated that state-of-the-art performance can also be achieved by the single kernel approach.

## Supporting Information

Tool S1 DDI_Extraction_Tool.
(ZIP)
